# Simultaneous integrated boost intensity-modulated radiotherapy for treatment of bone metastases: analysis of a breast cancer cohort

**DOI:** 10.2340/1651-226X.2025.42933

**Published:** 2025-05-19

**Authors:** Fabio Marazzi, Valeria Masiello, Alessandra Fabi, Stefania Manfrida, Barbara Corvari, Valentina Lancellotta, Ciro Mazzarella, Silvia Longo, Martina De Angeli, Francesca Moschella, Alba Di Leone, Armando Orlandi, Serena Bracci, Giuseppe Ferdinando Colloca, Mariangela Massaccesi, Luca Boldrini, Luca Tagliaferri, Emilio Bria, Riccardo Masetti, Gianluca Franceschini, Vincenzo Valentini, Maria Antonietta Gambacorta, Francesco Cellini

**Affiliations:** a Radioterapia Oncologica, Dipartimento di Diagnostica per Immagini, Fondazione Policlinico Universitario Agostino Gemelli IRCCS, Rome, Italy; b Unita Operativa Dipartimentale di Medicina di Precisione in senologia, Dipartimento di Scienze della salute della donna, del bambino e di sanita pubblica Fondazione Policlinico Universitario Agostino Gemelli IRCCS, Rome, Italy; c Chirurgia Senologica, Dipartimento di Scienze della salute della donna, del bambino e di sanità pubblica Fondazione Policlinico Universitario Agostino Gemelli IRCCS, Rome, Italy; d Oncologia Medica, Dipartimento di Dipartimento di Scienze Gastroenterologiche, Endocrino-metaboliche e Nefro-urologiche, Comprehensive Cancer Center. Fondazione Policlinico Universitario Agostino Gemelli IRCCS, Rome, Italy; e Istituto di Radiologia, Università Cattolica del Sacro Cuore, Rome, Italy; f Centro di Eccellenza Oncologia Radioterapica, Medica e Diagnostica per Immagini, Ospedale Isola Tiberina-Gemelli Isola, Rome, Italy; g Dipartimento di Scienze Mediche e Chirurgiche, Università Cattolica del Sacro Cuore, Rome, Italy

**Keywords:** breast cancer, SIB-IMRT, bone metastases, precision medicine

## Abstract

**Background:**

Bone metastases occur in up to 75% of metastatic breast cancer (MBC) cases. Advances in imaging now allow earlier detection, even during the oligometastatic phase. Radiotherapy (RT) is increasingly used in asymptomatic patients with ≤5 bone lesions, however standardised guidelines for dose and target volumes remain lacking. This study evaluates the outcomes of a simultaneous integrated boost (SIB) using intensity-modulated radiotherapy (IMRT) to deliver ablative doses to macroscopic bone lesions.

**Methods:**

This retrospective study analysed MBC patients treated with SIB-IMRT for bone metastases between January 2014 and January 2022. The primary endpoint was freedom from local progression (FFLP); secondary endpoints included disease progression after radiotherapy (DP-AR) and overall survival (OS). Subgroup analyses were performed according to age, immunophenotype, and line of therapy.

**Results:**

Among 954 patients treated with RT, 85 received SIB-IMRT (6–8 Gy per fraction, 5 fractions). Median follow-up was 41 months. Nineteen patients (22.4%) had a single bone metastasis, 23.5% were oligometastatic, and 54.1% were plurimetastatic. Median FFLP was 17 months; only 7% experienced local relapse at the SIB site. While DP-AR was 13.2 months, median OS reached 82.7 months. No significant correlation was found between local relapse and age, immunophenotype, or systemic therapy. Immunophenotype significantly influenced DP-AR (*p* = 0.002), while DP-AR and OS were not significantly associated with local progression.

**Interpretation:**

SIB-IMRT for bone metastases in MBC is feasible and effective, with encouraging local control and minimal toxicity. Prospective studies are warranted to optimise dose escalation and explore synergistic effects with systemic therapies.

## Introduction

In recent decades, the clinical management of metastatic breast cancer (MBC) has significantly evolved, leading to marked improvements in patient outcomes. Thanks to advances in diagnostic imaging, new technologies for local treatments, and novel targeted therapies, survival of patients with MBC is slowly but steadily improving [1], and the risk of death is decreasing by 1–2% each year [2]. Current guidelines are also being developed to define metastatic patients based on disease burden and expected prognosis [3, 4]. A recent classification of oligometastatic patients, published in 2020 by Guckemberger et al., classified different presentation of oligometastatic disease, based on a dynamic model that can present different therapeutic goals according to tumour active burden, time of revaluation, and response to therapies. Alongside this need for prognostic classification, local therapies are playing an increasingly important role in eradicating macroscopically visible disease. [5]. In particular, for breast cancer, over the years, there has been growing interest in the use of metastasis-directed therapy [6]. This approach is especially considered for patients with oligometastatic disease, more indolent immunophenotypes (such as luminal-like), or in cases where therapy is guided by specific drivers, such as BReast CAncer gene (BRCA) mutations or HER2 amplification [7]. An unmet need is also how to manage local therapies in patients who are oligopersistent or oligoprogressive from a previous plurimetastatic disease presentation, that could present intermediate prognosis.

Stereotactic-body radiotherapy (SBRT) is recognized as an effective and non-invasive local therapy for ablation of macro-metastatic disease. A 2018 study by Possanzini et al. [6], a reviewed SBRT in MBC cohorts and suggested it may play an important role in the management of these patients, potentially improving clinical outcomes with minimal toxicity. Furthermore, from the SABR-comet study, we know that patients with oligometastatic disease can benefit from stereotactic treatments, in particular in terms of overall survival (OS), as well as possible advantages of synergy with systemic therapies [8]. Nevertheless, despite these encouraging data, the optimal dose and target volumes in this setting remain uncertain. For this reason, we focused on the treatment of bone metastases in MBC patients using a particular SBRT technique, which involves the administration of a simultaneous integrated boost (SIB) to the bone lesion while concomitantly irradiating the entire bone compartment. Currently, data on the safety and efficacy of this specific approach are limited.

The aim of this study is to evaluate the clinical outcomes and toxicity profile of SIB-IMRT in MBC patients. To achieve this, we conducted a retrospective analysis assessing freedom from local failure and survival outcomes in MBC patients who underwent SIB-intensity modulated radiotherapy (IMRT) for bone metastases.

## Material and methods

### Patients selection

All patients referred to our department for radiotherapy (RT) evaluation, underwent an assessment for SIB-IMRT on bone lesions.

Inclusion criteria were:

- Histologically confirmed MBC (all patients underwent a biopsy if diagnosed as de novo metastatic, and in cases of metastatic relapse, a re-biopsy was performed).
- ECOG (Eastern Cooperative Oncology Group) performance status 0–2.
- Recent instrumental re-evaluation with computed tomography (CT) scan, positron emission tomography (PET)/CT scan, and/or magnetic resonance imaging (MRI) within the last month.
- No more than five bone lesions requiring RT for symptom relief or local control.
- No previous irradiation at the selected site.
- A minimum follow-up period of 6 months.

Exclusion criteria were:

- Patients with progressive plurimetastatic disease involving multiple sites (> 5 sites at the last evaluation).
- More than two contiguous bone compartments requiring irradiation.
- Patients scheduled for surgical intervention.

### Data collection

From time to enrolment, patients data were collected prospectively. In particular, data on patients’ characteristics (age at diagnosis, ECOG, pain symptoms before and after SIB-IMRT, antalgic therapy before and after SIB-IMRT), breast tumour characteristics (data of diagnosis, immunophenotype, stage at diagnosis, data of first metastasis, tumour bone burden at diagnosis, visceral metastases at diagnosis), data on systemic therapies ongoing (type of systemic therapy ongoing, denosumab administration, number of systemic therapies administered at the moment of SIB-IMRT prescription), data on SIB-IMRT administered (bone district treated, volumes prescribed, dose prescribed, data of SIB-IMRT ending), data on instrumental evaluation (imaging pre- SIB-IMRT, imaging post-SIB-IMRT), data on follow up and outcomes (local response to SIB-IMRT, data of local progression, data of systemic progression, data of last follow up, data of death). Toxicities from SIB-IMRT were reported according to Common Terminology Criteria for Adverse Events (CTCAE) v5.0 scale.

### SIB-IMRT technique description

**Figure 1:**
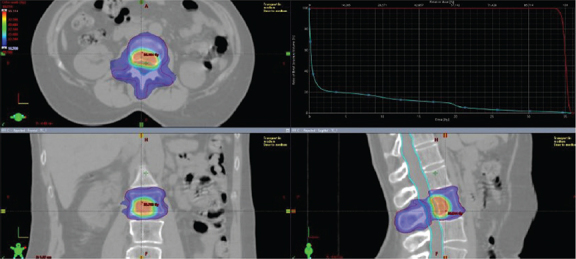
Treatment plan of a SIB-IMRT treatment. SIB-IMRT: simultaneous integrated boost-intensity modulated radiotherapy.

Patients underwent CT simulation with custom immobilization using Aquaplast® head masks and/or vacuum mattresses to ensure consistent positioning Target delineation mandatorily involved co-registration with diagnostic imaging, including contrast-enhanced CT, PET/CT, and MRI. Gross tumour volume (GTV) was defined as the visible lesion at diagnostic exams. There was not a dimensional criteria of macroscopic disease visible at instrumental exams used for contouring. ‘Macroscopic’ disease was identified as visible lytic or sclerotic or mixed lesion at II level (CT scan, PET-TC, MRI) used for delineation of planning target volume (PTV)1. Contouring defined two volumes: PTV1, including GTV plus 2-millimeters (mm) margin and PTV2, including entire bone compartment (for long and flat bones in the shoulder or pelvic girdle: the entire bone; for vertebrae: the entire vertebra) plus a 2 mm isotropic margin. For vertebral lesions, both spinal canal and spinal cord were contoured. Organ at risks were contoured on the basis of lesion’s site, and dose constraints were maintained according to American Association of Physicists in Medicine (AAPM) reports [9]. The planned doses ranged from 20 to 30 Gy to PTV2, with a number of fractions from 3 to 10 in accordance to clinical practice. Three different SIB doses were delivered to PTV1 (35, 40 and 50 Gy). PTV1 coverage was set at 95% of the prescribed dose at 95% of the defined volume. Major deviation for PTV2 was < 77% of the dose prescribed at 95% of the volume, while minor deviation was < 84% of the prescribed dose at 95% of the volume. For PTV1, major deviation was defined as < 79% of the prescribed dose at 95% of the volume, while minor deviation will be < 84% of the prescribed dose at 95% of the volume. PTV1 was normalized to 80% of the prescribed dose, which resulted in a dose gradient within the lesion, ranging from 120% to 125% of the prescribed dose. Reporting of dose prescription will be done according to International Commission on Radiological Units (ICRU) 83. A representative figure of treatment plan is reported in Figure 1. Treatment plan reporting was also according to ICRU 83.

### Endpoints

Primary endpoint of this study was freedom from local progression (FFLP), measured as time from the end of SIB-IMRT RT to first evidence of local progression at radiological (CT scan, PET, MRI) follow up. Patients underwent radiological exams every 6 months, except in case of clinical suspicious of progression. At radiological exams, local and systemic responses to treatments were classified according to RECIST v1.1 criteria [10] and/or PERCIST criteria [11]. Secondary endpoints were rate of disease progression after radiotherapy (DP-AR) and OS. DP-AR was measured as time from end of SIB-IMRT RT to first event of disease (local and/or systemic) progression at instrumental exams. Overall survival was measured as time from data of diagnosis to data of death or data of last follow up. Subgroup analysis (age, immunophenotype, line of therapy) were performed in order to correlate outcomes to possible prognostic factors.

## Results

From January 2014, among 954 MBC patients who underwent RT on metastatic lesions, 85 patients underwent SIB-IMRT RT. Patients’ characteristics and breast tumour characteristics are summarised respectively in Tables 1 and 2. Almost one third of patients (26 patients, 30.5%) were metastatic at diagnosis. While 67 patients (78,8%) were on first line systemic therapy, 11 patients (13%) were on second line, and 7 patients (8,2%) were on subsequent lines. Further, 44 patients (52%) were on Cyclin-Dependent Kinases (CDKs) 4/6 inhibitors therapy, 15 patients (18%) on anti-HER2 therapy, 11 patients (13%) on therapy with cytotoxic drugs, 14 patients (16%) on endocrine therapy, and 1 patient (1%) was on PARP inhibitors therapy. Furthermore, 39 patients (45.8%) had a co-adjuvant therapy with denosumab ongoing at the moment of SIB-IMRT administration.

**Table 1:** Patients’ characteristics.

Characteristic	Value
Age	Median 51 (Range 25–83)
ECOG Performance Status	
0	55 patients
1	26 patients
2	4 patients
NRS Scale Before SIB-IMRT	
0	66 patients
1–3	15 patients
4–6	11 patients
7–10	3 patients
Antalgic Therapy Before SIB-IMRT	
No therapy	77 patients
Non-opioid antalgic therapy	2 patients
Weak opioids therapy	1 patient
Strong opioids	5 patients
NRS Scale After SIB-IMRT (3 Months)	
0	82 patients
1–3	3 patients
4–6	0 patients
7–10	0 patients
Antalgic Therapy After SIB-IMRT (3 Months)	
No therapy	81 patients
Non-opioid antalgic therapy	1 patient
Weak opioids therapy	1 patient
Strong opioids	2 patients

**Table 2:** Disease characteristics.

Histology	
Ductal	52 pts
Lobular	13 pts
Other [special types]	20 pts
Immunophenotype	
Luminal A	16 pts
Luminal B	45 pts
HER2+	15 pts
TNBC	9 pts
Stage at diagnosis	
I	17 pts
IIA	12 pts
IIB	7 pts
IIIA	6 pts
IIIB	14 pts
IIIC	3 pts
IV	26 pts
Tumour bone burden at diagnosis	
Single bone metastasis	19 pts (22.4%)
Oligometastatic	20 pts (23,5%)
Plurimetastatic	46 pts (54,1%)
Visceral metastasis at diagnosis	
No	46 pts (54,1%)
Yes	39 pts (45,9%)

HER: Human Epidermal Growth Factor Receptor; pts: patients; TNBC: Triple Negative Breast Cancer.

### SIB-IMRT RT treatments

On 85 patients, a total of 125 lesions underwent SIB-IMRT, according to the following treatment schedules:

1. PTV1 (macroscopic bone lesion(s): 40–30 Gy in 5 fractions (fr); PTV2 (entire bone compartment) 20 Gy in 5 fr.
2. PTV1 (macroscopic bone lesion(s): 30 Gy in 3 fr; PTV2 (entire bone compartment) 21 Gy in 3 fr.
3. PTV1 (macroscopic bone lesion(s): 50 Gy in 10 fr; PTV2 (entire bone compartment) 30 Gy in 10 fr.

Eighty-five lesions were irradiated according to schedule A, 16 lesions according to schedule B, and 24 lesions according to schedule C. Among lesions who underwent schedule A, 25 lesions received 40 Gy on PTV1, 47 lesions received 35 Gy on PTV1, and 13 lesions received 30 Gy on PTV1. PTV1 also included multifocal sites of disease visible at instrumental exams in the same bone compartment. Forty-five patients had a basal PET for contouring, 19 patients had a CT scan with iodine contrast, 15 patients were contoured on MRI imaging, 4 patients on both PET and MRI, 1 patient had both PET and CT with iodine contrast, and 1 patient had both MRI imaging and CT with iodine contrast.

Six patients received treatment on shoulder girdle lesions, 46 patients on vertebrae, 23 patients on pelvic girdle, 8 patients on both vertebrae and pelvic girdle, 1 patient on both shoulder girdle and vertebrae, and 1 patient on both shoulder and pelvic girdle.

### Survival outcomes

**Figure 2:**
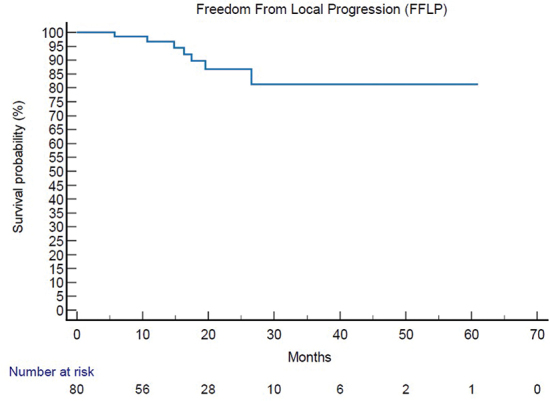
Kaplan Meier analysis of primary endpoint [Freedom From Local Progression – FFLP]. FFLP: Freedom From Local Progression.

**Figure 3:**
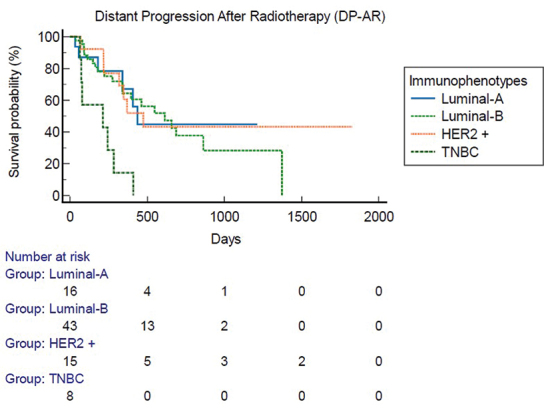
Distant progression-after radiotherapy [DP-AR] with subgroup analysis for immunophenotypes.

Mean follow up was 41 months (6–61.5 m). At instrumental exams 6 months after SIB-IMRT, response on irradiated sites was complete response on 31 patients, partial response on 19 patients, stable disease on 34 patients, and progression disease on 1 patient. Among the total patients, 53 patients underwent re-evaluation PET after SIB-IMRT with 33 patients with a complete metabolic response, 13 patients with a partial metabolic response, 6 patients with a stable metabolic response, and 1 patient with progressive metabolic response. FFLP was 17 months (95% CI 3.2–61.5 m) (Figure 2), And FFLP was not related to RT regimen administered (*p* 0.73). Only 6 patients (7%) had local relapse. DP-AR was 13.2 m (95% CI 3.1–56.9 m). OS was 82.7 m (95% CI 10.6–343 m). Local-relapse was not associated with age, immunophenotype or systemic line ongoing. Among secondary outcomes, DP-AR resulted associated to immunophenotype (*p* 0.002) (Figure 3). DP-AR and OS were not significantly associated with local relapse (respectively *p* 0.148 and *p* 0.4). About safety and tolerance, event of pain flare during SIB-IMRT had an incidence of 37% and were managed with steroids administration, while about late toxicity, no events of bone fracture were observed during follow up.

## Discussion

Metastatic Breast Cancer is a challenging setting of care and thanks to implementation of treatments, we are assisting to a progressive implementation of outcomes in the last years [2]. In particular, among the spectrum of MBC, oligometastatic breast cancer seems to have better prognosis probably due to its unique molecular signature [12, 13] and data on SBRT administration in this setting are encouraging in terms of progression-free survival (PFS) [14–18].

**L**imitations of the study include the absence of randomisation and the heterogeneous nature of the cohort, which includes patients with different primary tumours. Additionally, there is a lack of clear data and standardised indications for locoregional therapy in the setting of oligorecurrent or oligoprogressive disease.

Furthermore, while this study provides insights into dose escalation, it does not establish definitive guidelines for optimal dose and volume selection, which may limit its immediate clinical applicability. A comparison with other standard RT regimens, such as palliative 8 Gy or 30 Gy in 10 fr, is not included, and this would be valuable in contextualising the local PFS results.

Another limitation is the retrospective nature of the analysis, which may introduce selection bias. Additionally, patient-reported outcomes (PROs), such as pain relief and quality of life, were not assessed, which could have provided a more comprehensive understanding of treatment benefits.

Lastly, while the study highlights the potential synergy between RT and systemic treatments, it does not clarify whether systemic therapies were administered concurrently with RT or paused to minimise potential toxicities, such as gastrointestinal and hematologic side effects. This information is critical for assessing the safety and feasibility of combining these therapies in routine clinical practice.

### Dose and volumes considerations

In recent decades, clinical trials have been increasingly aimed at responding to an unmet need for ever clearer data on what are the ideal volumes and doses for SBRT treatments on metastatic lesions [19–23]. Marazzi et al. proposed an algorithm for local treatment of bone metastases to choice dose and volumes for RT on bone metastases according to prognosis [7]. According to this literature review, in case of patients with an intermediate-good prognosis, an ablative RT treatment (Biological Equivalent Dose (BED) > 75 Gy) should be proposed to patients.

### Literature review of SIB-IMRT applied to bone metastases

SIB found a recent application in RT treatments; in general, it was originally applied in H&N and prostate cancer dose escalation [24–26]. In recent years, several in silico studies have evaluated the feasibility of applying SIB-IMRT to bone metastases. For example, Lee et al. compared IMRT and Volumetric Modulated Arc Therapy (VMAT) techniques for the planning and delivery of SIB-IMRT in vertebral lesions. PTV coverage was equivalent with both techniques, but VMAT was superior in terms of spinal cord sparing (*p* 0.04) and of mean delivery times (3,5 vs. 10.5 min), reducing risk of acute and late toxicities and of target missing [27]. A literature review of clinical data is reported in Table 3. Very few data on small cohorts (less than 100 patients) are reported in literature. These cohorts included mixed solid tumours, but encouraging data on good local control after 2 years from RT with low rates of toxicities are reported. Ongoing trials that actually are enrolling patients to test SIB-IMRT on bone metastases are:

**Table 3:** Literature review of SIB-IMRT evidences on bone metastases.

Author	N° pts Pts characteristics	Dose and volumes	Pain control	Local control	OS	Toxicities
Murai T, 2014 **[****32****]**	30 pts Different solid tumours (MBC 5 pts tot) Only vertebral metastases	Good Prognosis: 48 Gy/16 Fr to PTV1, 44 Gy/16 Fr to PTV2, and 40 Gy/16 Fr to PTV3 Less favourable prognosis: 40 Gy/8 Fr to PTV1, 36 Gy/8 Fr to PTV2, and 32 Gy/8 Fr to PTV3	95% at 2 m from SIB-IMRT	1-y 84% (R 70–100%)	6-m 60% 12-m 40%	Dermatitis G2 1% Dermatitis G1 6% Enteritis G3 1%
Farooqi A, 2019 **[****33****]**	12 pts Different solid tumours (RCC 73%, 1 pts with breast angiosarcoma) Only vertebral metastases	GTV and CTV were prescribed 40 Gy and 30 Gy, respectively, in 10 fractions using step-and-shoot IMRT (13 sites) or VMAT (2 sites)	NA	1-y 93%	1-y 58%	Fatigue 58% Erythema 25% Nausea 16,6%
Jacobs JD, 2019 **[****34****]**	42 pts Different solid tumours [prostate (36%), gastrointestinal (24%), and lung (24%)] Both bone and nodal metastases	50 Gy to the PTVboost and 30 Gy to the PTVelect simultaneously in 10 fractions	1-y 94%	1-y 90%	1-y 88,1%	Fatigue 55% Gastrointestinal G1-2 42% Neutropenia G3 3%
Shenker RF, 2022 **[****35****]**	101 pts Different solid tumour [prostate (37%), lung (15%), and breast (7%)] Randomised to SIB-IMRT technique at 90 sites (53% nodal and 47% osseous) or SBRT at 46 sites (13% nodal and 87% osseous)	SIB-IMRT cohort 50 Gy to the treated metastases and 30 Gy to the elective PTV in 10 fractions SBRT cohort doses ranged from 18 Gy in 1 fraction (22%) to 50 Gy in 10 fractions (50%)	86% with SIB-IMRT 82% with SBRT	2-y SIB-IMRT 98% 2-y SBRT 87%	1-y 88% in both groups	Acute toxicities G3 0% in both group Late toxicities G3 4,5% in SIB-IMRT cohort vs. 0% SBRT cohort
Potkrajcic V, 2022 **[****36****]**	24 pts Different solid tumour [prostate 45,8%, genito-urinary 20,9%, others 33%]	30/40 Gy with simultaneous integrated boost (SIB) in 10 fractions on bone metastases	NA	1-y 90%	NA	Acute toxicities G1 36%
Floretz MA, 2023 **[37]**	58 pts Different solid tumours	GTV and CTV were prescribed 40 and 30 Gy in 10 fractions, respectively	82%	1-y 88% 2-y 74%	1-y 64% 2-y 45%	13% late fracture events

CTV: clinical target volume; FR: fraction; G: grade; Gy: grey; GTV: gross tumour volume; pts: patients; y: year; IMRT: intensity modulated radiotherapy; m: months; MBC: metastatic breast cancer; N: number; NA: not available; OS: overall survival; PTV: planning target volume; r: range; SBRT: stereotactic body radiotherapy; SIB: simultaneous integrated boost; VMAT: Volumetric Modulated Arc Therapy.

– NCT02832765 [28], a single-centre, prospective, randomised controlled trial, to test four treatment arms planned: IMRT with 30 Gy in 10 fr, IMRT with 30 Gy in 10 fr and SIB to 40 Gy, IMRT with 20 Gy in 5 fr, and IMRT with 20 Gy in 5 fr and SIB to 30 Gy in 5 fr will be compared. Primary endpoint is local control.
– NCT03597984 [19], a phase 3, open-label, multicentric trial randomised patients to test standard conventional RT involving 4 Gy × 5 fr to the whole involved vertebra or SBRT by intensity modulated RT with simultaneous integrated boost (IMRT-SIB) involving 7 Gy × 3 fr to the whole involved vertebra + 10 Gy × 3 fr on the macroscopic lesion. Primary endpoint is overall pain reduction.

### Outcomes and application for MBC

In this study, results on feasibility, tolerance, and clinical outcomes of a SIB-IMRT administered on bone lesions from MBC were presented. To our knowledge, it is the first such large cohort of patients with MBC treated with SIB-IMRT presented in the literature. All the outcomes in terms of safety and tolerance are in line with data reported in literatures. Mean FFLP was 17 months, this result is more than what is reported by other SIB-IMRT cohorts in which probably patients with different type of solid tumour are enrolled, particularly with the worse prognosis of breast cancers.

This study presents some limitations due to retrospective analysis and absence of a control arm. Although this technique is not extensively used at present and there are still not specific guidelines on its application, it has been shown to improve the therapeutic ratio and may improve local control, in addition to shortening the course of RT [29]. Surely for breast cancer, at the presented time we had the possibility of different systemic therapies, some of them with very long PFS when administered on first line [4]. From literature, we know that there are also some possible synergistic effects of combining systemic therapies and RT. For example, pre-clinical data suggest a potential synergy between RT and CDK4/6 inhibitors, in particular the addition of RT to palbociclib, have shown to increase in the DNA damage marker, γH2AX and the apoptotic marker [30]. Another interesting mechanism of possible synergistic effects is represented by association of PARP inhibitors and SBRT. In fact, many pre-clinical models have described that the combination of PARP inhibitors and RT can intensify DNA damage, and promote cancer cell death [31]. For these reasons, the possibility to administer a dose-escalating treatment on macroscopic disease, with few number of fractions, to enhance synergistic effects with systemic therapies can be considered especially for patients with low tumour burden and during first and second lines of treatments.

## Conclusion

This study confirms the feasibility and effectiveness of SIB-IMRT for bone metastases in MBC patients, achieving a 93% local control rate with a mean FFLP of 17 months. These results align with existing safety and tolerance data.

However, limitations include its retrospective design, absence of a control arm, and lack of standardised systemic therapy administration. Additionally, the optimal dose and volume selection remain undefined, and PROs were not assessed.

Despite these limitations, SIB-IMRT may improve the therapeutic ratio by enhancing local control while shortening treatment duration. Further prospective trials are needed to define optimal dose-escalation strategies and evaluate synergistic effects with systemic therapies to improve DP-AR and overall outcomes.

## Data Availability

The data supporting the conclusions of this study are available from the corresponding author upon reasonable request. Owing to patient privacy and ethical considerations, the dataset is not publicly available.
